# Supplementary material for: A quantitative analysis of the interplay of environment, neighborhood, and cell state in 3D spheroids
Source: Mol Syst Biol. 2020 Dec 26;16(12):e9798. doi: 10.15252/msb.20209798 (PMC7765047; doi:10.15252/msb.20209798)
Supplement: Supplementary file 1 — Expanded View Figures PDF [file MSB-16-e9798-s001.pdf]

Expanded View Figures

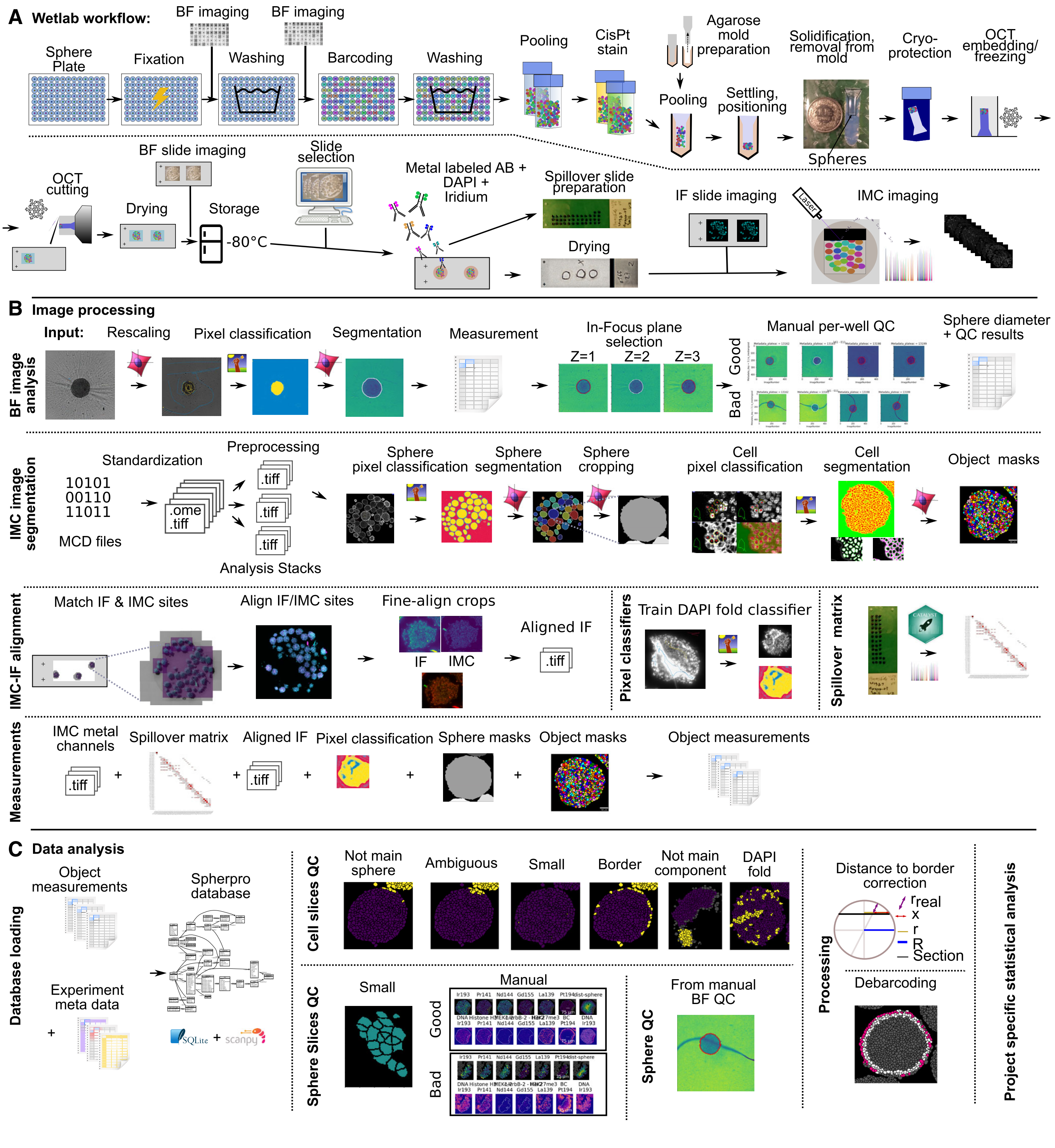

Figure EV1. Spheroid barcoding and analysis workflow.

A–C Schematic illustration of the complete Wet Lab (A), image processing (B), and data analysis (C) workflow.

 © 2020 The Authors

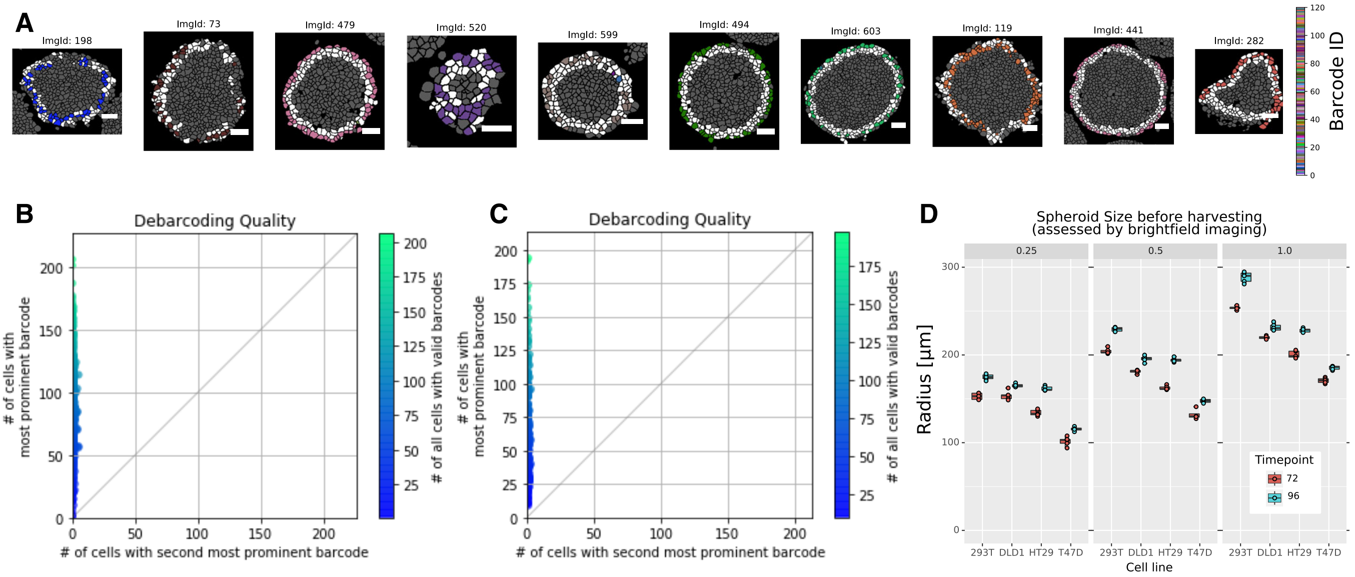

Figure EV2. Debarcoding results.

A Representative barcoding results on the cell level. Cells were debarcoded by determining whether the threshold barcode channels corresponded to a valid barcode (colored cells) or not (white cells). Cells outside of the border of spheres were not considered for debarcoding (gray cells). The sphere section barcode assignment required that the majority barcode was present in at least ten cells and in more than twice the number of cells than the second-most abundant barcode. The white scale bar indicates 50 µm.

B Number of cells with the most prevalent barcode plotted versus the number of cells with the second-most prevalent barcode per sphere from a dataset of 120 barcoded spheres.

C Number of cells with the most prevalent barcode plotted versus the number of cells with the second-most prevalent barcode per sphere from a dataset of 360 barcoded spheres.

D Spheroid size of individual spheres at the indicated growth conditions: Each facet represents a relative cell seeding number, and color indicates the time point. Sphere radius was determined by image segmentation of bright-field images of intact spheres. Five technical replicate spheres were seeded in separate wells from the same cell dilution series. The box plot represents the 25th and 75th percentiles of the replicates, and the central line indicates the median.

© 2020 The Authors

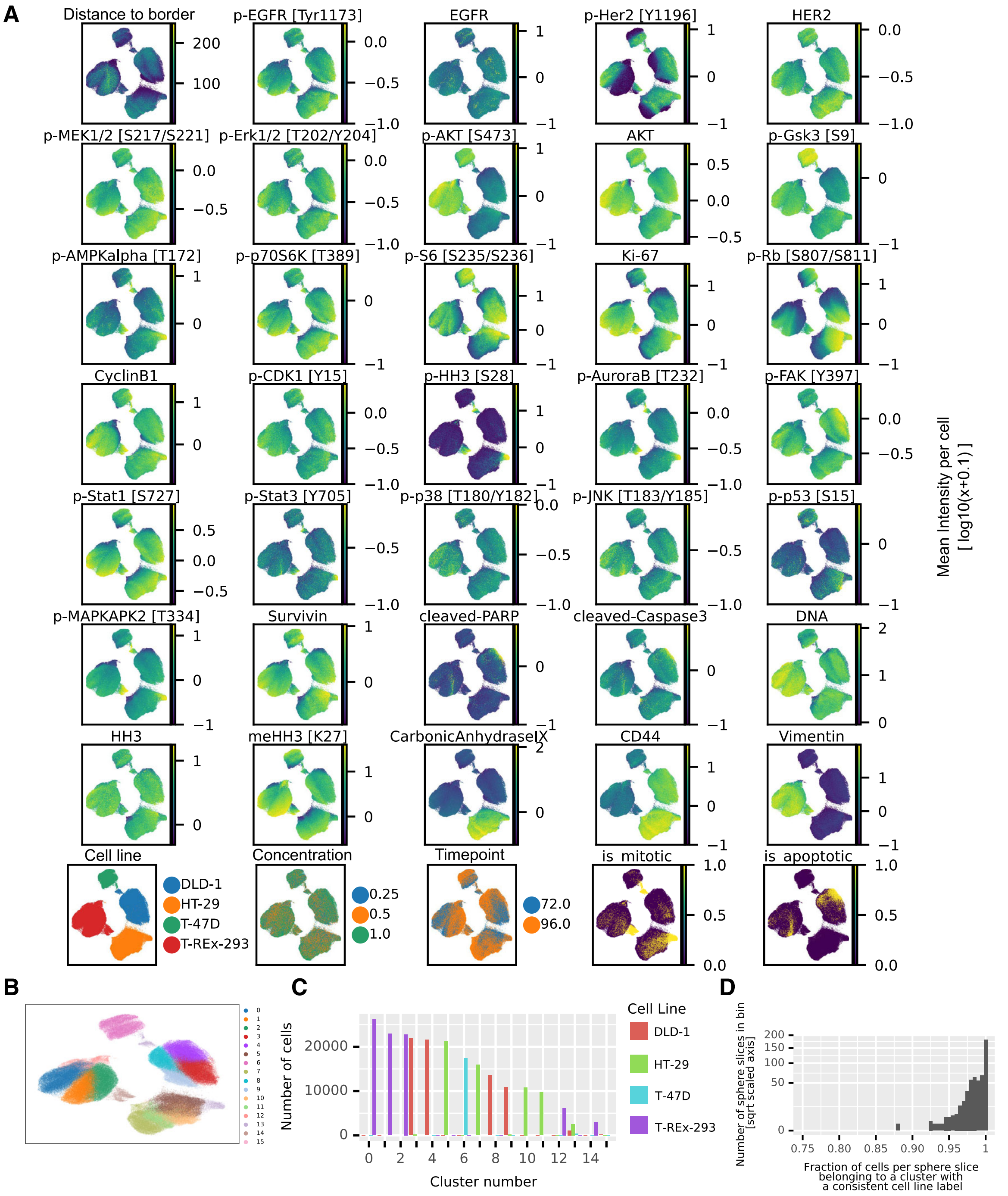

Figure EV3. Cell line marker analysis.

A Nonlinear dimensionality reduction with UMAP (McInnes *et al*, 2018) visualizes average marker levels across the unperturbed cell line dataset. The panels show marker levels with colors indicative of log10(MeanIntensity + 0.1) per marker. The first three panels of the bottom row visualize metadata mapped on the cells after barcoding. The two last panels indicate apoptotic (cleaved PARP positive) and mitotic (p-HH3 positive) clusters after individually clustering cell lines on cell cycle and apoptotic markers using the Leiden clustering (Traag *et al*, 2019).

B Clustering of the cell slice data using the Leiden clustering (Traag *et al*, 2019).

C Number of cells from each cell line per cluster.

D Fraction of cells per sphere slice where the debarcoded sphere slice label was identical to the most abundant cell line label present in the cluster.

 © 2020 The Authors

Figure EV4. Correlation analysis results for the other three cell lines.

A–C Correlation heat maps as in Fig 2D for the other three cell lines.

D Selected growth signaling and cell-cycle markers plotted as a function of distance to border for spheres of all cell lines (96 h growth, ca 200 μm diameter). Color scale: centered log10(x + 0.1) intensity.

© 2020 The Authors

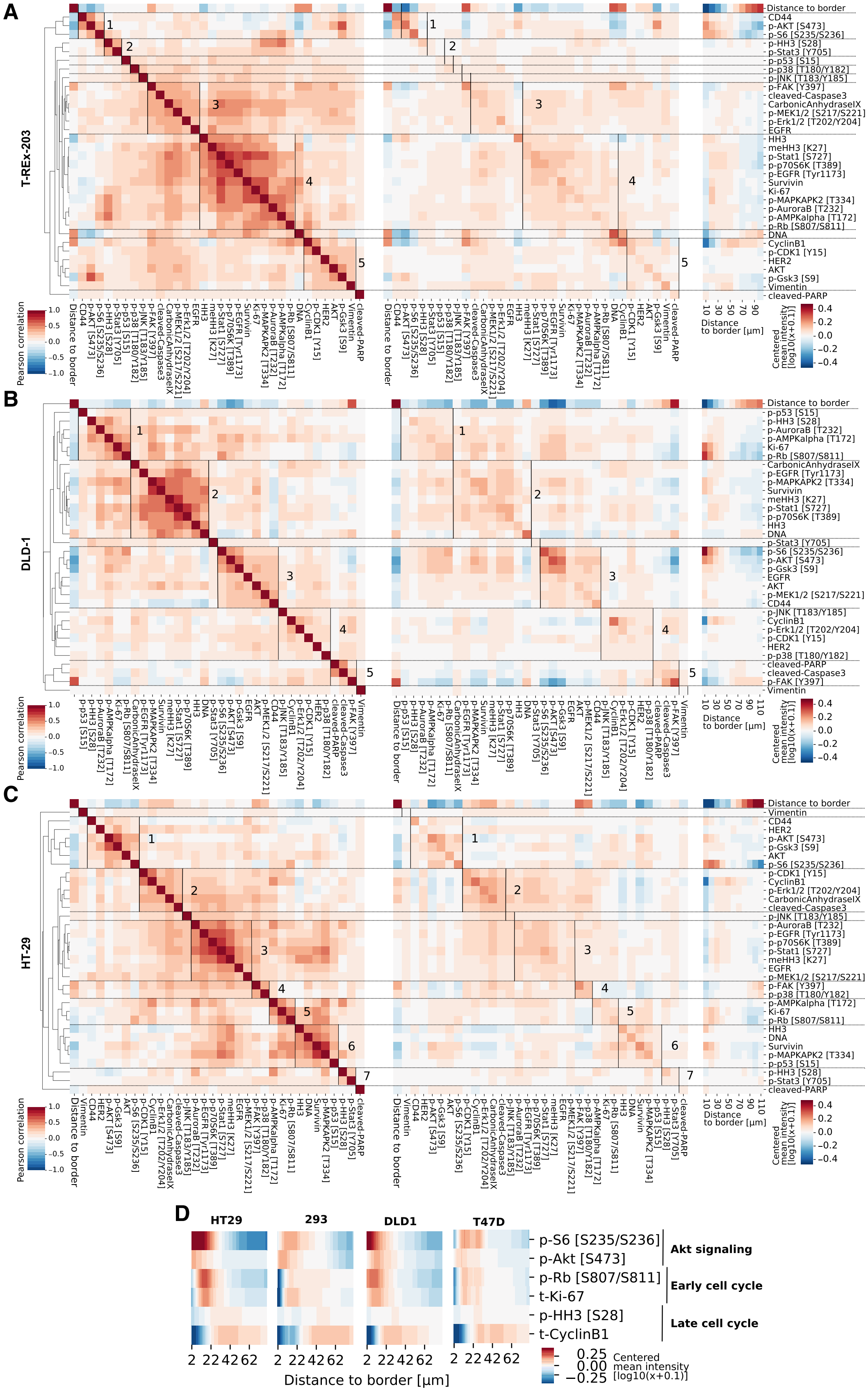

Figure EV4.

 © 2020 The Authors

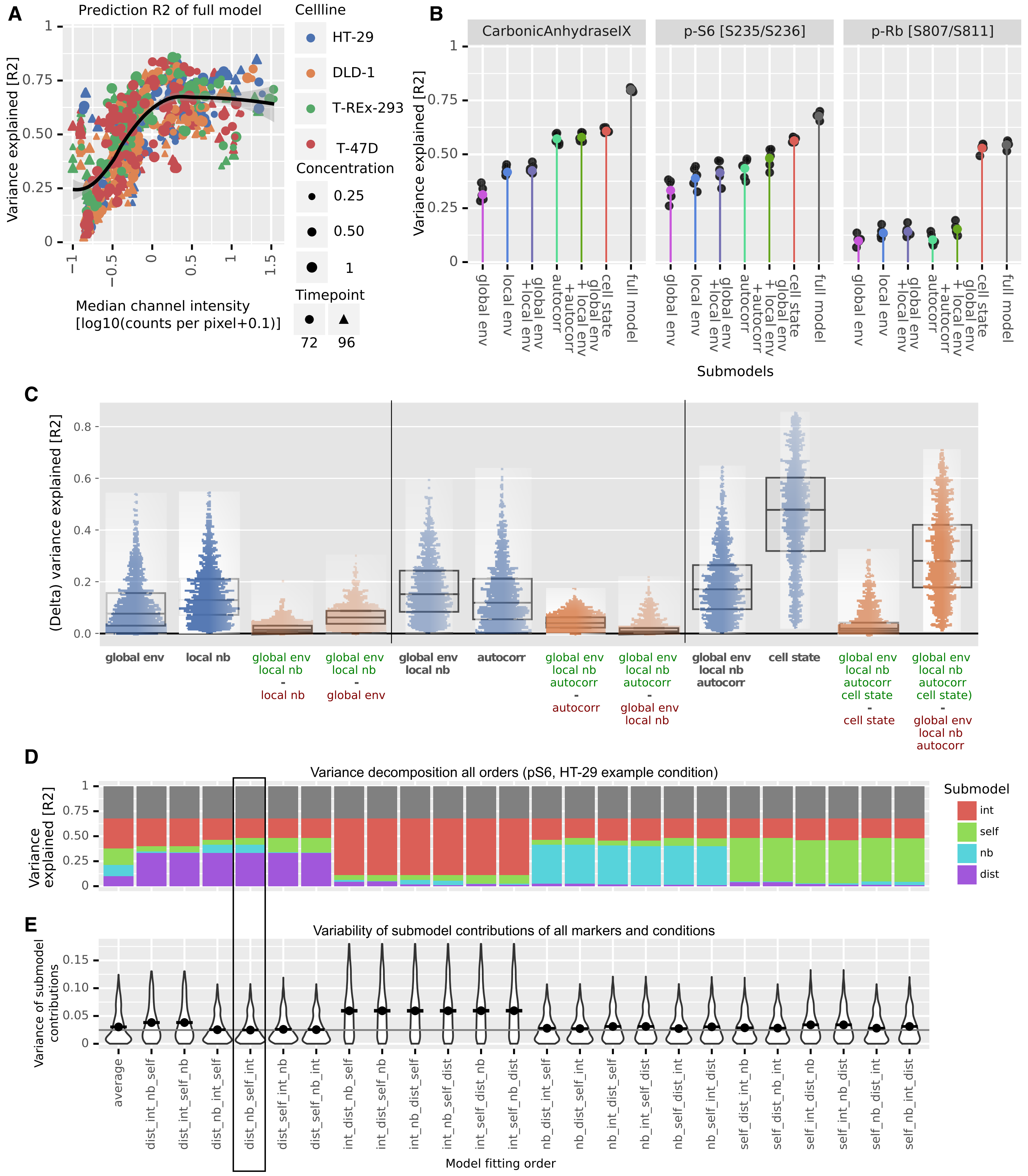
A
Prediction R2 of full model
Variance explained [R2]
Median channel intensity
[log10(counts per pixel+0.1)]
Cellline
HT-29
DLD-1
T-REx-293
T-47D
Concentration
0.25
0.50
1
Timepoint
72
96
B
CarbonicAnhydraseIX
p-S6 [S235/S236]
p-Rb [S807/S811]
Variance explained [R2]
global env
local env
global env +local env
autocorr
+autocorr + local env global env
cell state
full model
Submodels
C
(Delta) variance explained [R2]
global env
local nb
global env local nb - local nb
global env local nb - global env
global env local nb
autocorr
global env local nb autocorr - autocorr
global env local nb autocorr - global env local nb
global env local nb autocorr
cell state
global env local nb autocorr cell state - cell state
global env local nb autocorr cell state) - global env local nb autocorr
D
Variance decomposition all orders (pS6, HT-29 example condition)
Variance explained [R2]
Submodel
int
self
nb
dist
E
Variability of submodel contributions of all markers and conditions
Variance of submodel contributions
average
dist_int_nb_self
dist_int_self_nb
dist_nb_int_self
dist_nb_self_int
dist_self_int_nb
dist_self_nb_int
int_dist_nb_self
int_dist_self_nb
int_nb_dist_self
int_nb_self_dist
int_self_dist_nb
int_self_nb_dist
nb_dist_int_self
nb_dist_self_int
nb_int_dist_self
nb_int_self_dist
nb_self_dist_int
nb_self_int_dist
self_dist_int_nb
self_dist_nb_int
self_int_dist_nb
self_int_nb_dist
self_nb_dist_int
self_nb_int_dist
Model fitting order

Figure EV5.

◀ **Figure EV5. Additional analyses of variance explained by linear model.**

A Variance of each marker explained by the full linear model plotted versus median marker level. Data are shown for all cell lines, growth conditions, and growth times. The black line indicates a smooth conditional mean fit using LOESS with the gray shading indicating the 95% confidence interval of the fit.

B Variance explained by models with different submodules (x-axis) for three example markers for the HT-29 growth condition used in Fig 2C. Colored points indicate the variability explained over all cells of these growth conditions. Black points indicate the variability explained for cells of each of the five individual sphere replicates.

C Variance of each marker explained by various submodels (blue dots, legend: model elements) or difference in variance explained between submodels (orange dots, legend: subtraction of variability of model with red modules from variability of model with green modules). Each data point represents a single marker from one of the 24 growth conditions (4 cell lines, 2 time points, 3 concentrations). Box plot boxes represent the 25th and 75th percentiles of the data, the central band, and the median.

D Bar plots as in Fig 4 but calculated from all possible orders of adding the submodules. The order is indicated in the x-axis label. The bar marked “average” shows the contributions averaged over all model orders. Modules: dist: global environment, nb: local neighborhood, self: autocorrelation, int: cell state. The data shown are for the marker pS6 of the HT-29 growth condition used for Fig 2C (96-h growth, largest size). The order favored by our conceptual model of cells interacting in tissue (dist, nb, self, int) is highlighted.

E Variance of marginal contributions of submodels (colored areas in bar plot (D)) when adding them according to the indicated order over all markers and conditions (violins). The point indicates the mean variance for a given order and the line indicates the minimal average variance, achieved by the dist, nb, self, int order.

© 2020 The Authors

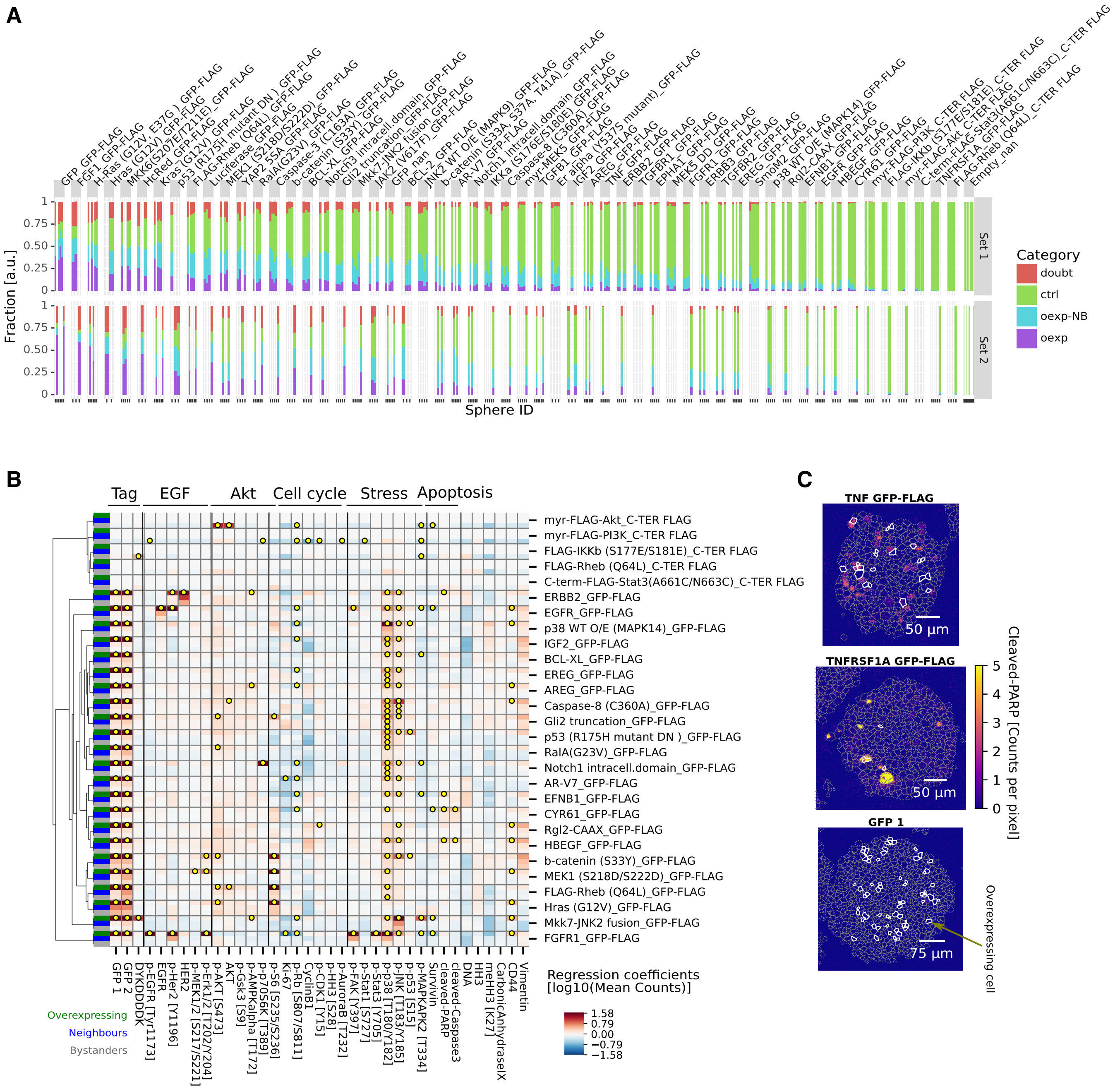

Figure EV6. Additional overexpression results.

A Fraction of overexpressing (violet), neighbors (blue), bystanders (green), or not assigned cells (red) for each sphere replicate (bar) per overexpression construct (facet). Constructs were sorted by average fraction of overexpressing cells.

B Matrix of overexpression constructs with intracellular effects only (rows) versus all effects on markers (columns) in cells classified as intracellular (green), neighborhood (blue), and bystander (gray) (indicated in column at the far left). Yellow dots indicate significant effects ($P < 0.01$, $q < 0.1$, FC > 20%, test: t-statistics for linear mixed-effects model coefficients using Satterthwaite's method for denominator degrees of freedom).

C Example IMC images of effects on cleaved PARP in spheroids with cells that overexpress TNF, TNFRSF1A, or GFP control. Color indicates counts per pixel. Gray dashed lines indicate segmentation mask borders, and white lines mark cells identified as overexpressing.

© 2020 The Authors
